# The short-term effects of sedentary behaviour on cerebral hemodynamics and cognitive performance in older adults: a cross-over design on the potential impact of mental and/or physical activity

**DOI:** 10.1186/s13195-020-00644-z

**Published:** 2020-06-22

**Authors:** Carlijn M. Maasakkers, René J. F. Melis, Roy P. C. Kessels, Paul A. Gardiner, Marcel G. M. Olde Rikkert, Dick H. J. Thijssen, Jurgen A. H. R. Claassen

**Affiliations:** 1 Department of Geriatrics/Radboud Alzheimer Center, Radboud Institute for Health Sciences, Radboud University Medical Center, Nijmegen, The Netherlands; 2 Department of Medical Psychology/Radboudumc Alzheimer Center, Donders Institute for Brain, Cognition and Behavior, Radboud University Medical Center, Nijmegen, The Netherlands; 3 Center for Cognition, Donders Institute for Brain, Cognition and Behavior, Radboud University, Nijmegen, The Netherlands; 4 Centre for Health Services Research, Faculty of Medicine, The University of Queensland, Brisbane, Australia; 5 Department of Geriatrics/Radboud Alzheimer Center, Donders Institute for Brain, Cognition and Behavior, Radboud University Medical Center, Nijmegen, The Netherlands; 6 Department of Physiology, Radboud Institute for Health Sciences, Radboud University Medical Center, Nijmegen, The Netherlands; 7 Research Institute for Sport and Exercise Science, Liverpool John Moores University, Liverpool, UK

**Keywords:** Cerebral vasculature, Cognitive decline, Dementia, Physical inactivity, Sitting

## Abstract

**Background:**

Sedentary behaviour might be a potential risk factor for cognitive decline. However, the short-term effects of sedentary behaviour on (cerebro) vascular and cognitive performance in older people are unknown.

**Methods:**

We used a cross-over design with 22 older adults (78 years, 9 females) to assess the short-term hemodynamic and cognitive effects of three hours uninterrupted sitting and explored if these effects can be counteracted with regular (every 30 min) two-minute walking breaks. In addition, we investigated if low versus high mental activity during the three hours of sitting modified these effects. Before and after each condition, alertness, executive functioning, and working memory were assessed with the Test of Attentional Performance battery. Additionally, cerebral blood flow velocity (Transcranial Doppler) and blood pressure (Finapres) were measured in rest, and during sit-to-stand and CO_2_ challenges to assess baroreflex sensitivity, cerebral autoregulation, and cerebral vasomotor reactivity.

**Results:**

No short-term differences were observed in cognitive performance, cerebral blood flow velocity, baroreflex sensitivity, cerebral autoregulation, or cerebral vasomotor reactivity across time, or between conditions. Blood pressure and cerebrovascular resistance increased over time (8.6 mmHg (5.0;12.1), *p* < 0.001), and 0.23 in resistance (0.01;0.45), *p* = 0.04). However, these effects were not mitigated by mental activity or by short walking breaks to interrupt sitting.

**Conclusions:**

In older individuals, three hours of sitting did not influence cognitive performance or cerebral perfusion. However, the sitting period increased blood pressure and cerebrovascular resistance, which are known to negatively impact brain health in the long-term. Importantly, we found that these effects in older individuals cannot be mitigated by higher mental activity and/or regular walking breaks.

**Trial registration:**

Clinical trial registration URL: https://www.toetsingonline.nl/. Unique identifier: NL64309.091.17. Date of registration: 06–02–2018.

## Background

The mechanisms behind the neurodegenerative processes that lead to late-onset Alzheimer’s disease are not clearly understood, and all recent drug trials failed to deliver a cure [1–3]. Epidemiological evidence suggests that physical inactivity is the strongest risk factor in the USA and Europe, responsible for approximately 20% of all Alzheimer’s disease cases [4]. Part of this increased risk might be explained by sedentary behaviour (SB; defined by the Sedentary Behaviour Research Network as “Any waking behavior characterized by an energy expenditure ≤1.5 metabolic equivalents (METs), while in a sitting, reclining or lying posture” [5]. SB, independent from lack of physical exercise, is a highly prevalent behaviour and could be a feasible target in older adults to slow cognitive decline [6]. Epidemiological observational studies, however, did not find evidence for an association between total SB and global cognitive decline over a mean follow-up range of 2.0–8.1 years [7], which contrasts with an earlier review suggesting such an association [8]. Detailed studies on underlying short- and long-term mechanisms of the effects of SB on the brain are therefore needed.

SB has been linked to multiple cardiovascular risk factors [9], which in turn are known to be associated with dementia. Experimental studies in young men showed an increase in diastolic blood pressure, decrease in peripheral artery blood flow, increase in peripheral resistance, and impaired vascular function after prolonged sitting [10, 11]. Since distinct mechanisms regulate peripheral versus cerebral hemodynamics, the question rises if these effects of SB on the peripheral vasculature may equally apply to the cerebral vasculature. Since normal cerebrovascular function is essential for brain health and cognition [12], repeated short-term cerebrovascular dysfunction induced by SB may contribute to long-term neurodegenerative mechanisms that underly dementia.

Indications for the existence of SB-induced cerebrovascular dysfunction come from studies reporting reduced cerebral blood flow (CBF) velocity after four [13] or eight hours of prolonged sitting [14], in respectively desk workers and older adults. Moreover, during a continuously seated workday a midday dip in CBF velocity was seen in working adults [15]. In addition, a cross-sectional study in older adults found an association between higher amounts of daily life SB and lower CBF in frontal brain regions [16]. This reduced CBF contrasts with the idea that CBF is adequately maintained by the combined actions of the baroreflex system and cerebral autoregulation (CA) [17]. Specifically, CA operates through cerebral vasoconstriction to counteract increases in BP, and cerebral vasodilation to counteract reductions in BP, to prevent brain hyper- and hypoperfusion. However, in extreme models of deconditioning such as bed-rest experiments, a blunting of the baroreceptor reflexes [18] and a reduced CBF were observed [19]. These experiments include long periods in which, in the absence of gravity, the baroreflex and CA systems are not or only minimally challenged. Short-term variability in arterial pressure and CBF appear to be cerebroprotective, for instance, via nitric oxide (NO) production that reduces peripheral vascular resistance [20]. Theoretically, prolonged sitting may equally result in reduced gravitational challenges to these systems, and in the short term impair these cerebroprotective effects, negatively affecting brain health and cognitive performance in the long term. Limited evidence is currently present on whether SB impairs cerebrovascular health in older individuals similar to healthy young populations.

Previous work revealed that SB comes in various forms, which may differentially affect cerebrovascular health [21]. For example, CBF and oxygenated haemoglobin levels increase when mental activity is higher [22, 23]. Additionally, the decrease in CBF could be mitigated by two minute light-intensity walking breaks every 30 min [13]. Walking or standing for 90 min spread out over a six hour seated working day, even resulted in improved cognitive performance in young adults [24]. The question therefore rises whether short-term exposure to SB alters cerebral hemodynamics and cognitive performance in an older population, and if these potential effects are mitigated by mental activity and/or regular physical activity breaks. Following previous work in young participants, we hypothesised that SB lowers cerebrovascular health and cognitive performance, which can be mitigated by higher mental or physical activity. We performed a cross-over clinical trial in 22 older adults of 70 years and over, which allowed us to compare uninterrupted sitting with interrupted sitting, both in combination with low versus higher mental activity on cerebrovascular health and cognition.

## Methods

### Participants

We recruited 22 older adults between the ages of 70 and 90 from research databases with participants who had given permission to be contacted (Supplement 1). Inclusion criteria for participation were a score of at least 24 on the Montreal Cognitive Assessment (MoCA) to exclude people with severe cognitive impairment, and the ability to sit for three hours without interruptions. Exclusion criteria were a diagnosis of dementia or mild cognitive impairment (MCI), a history of familial early-onset dementia, a past history of brain damage (including trauma, stroke or serious neurological disorder), a diagnosis or drug use for any major psychiatric disorders, drug use that affected alertness, or being vigorously physically active for more than three hours per week. All participants signed an informed consent and the study was approved by the institutional review board (CMO, Arnhem-Nijmegen), and conducted in accordance with the Declaration of Helsinki. The study was registered at the Dutch clinical trial registration Toetsingonline (NL64309.091.17).

### Design

**Fig. 1:**
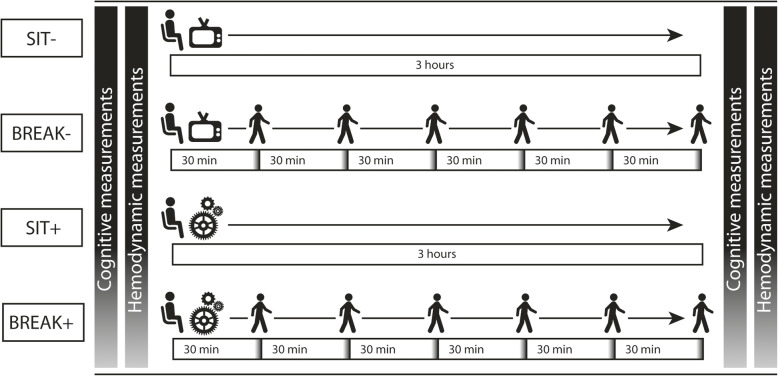
Study Design. Study design, with four conditions shown horizontally. During SIT- people stayed seated for three hours while watching television. In the BREAK- condition people interrupted their television watching with two-minute walking breaks. The SIT+ and BREAK+ condition are similar to the SIT- and BREAK- conditions, except for mental activity, which was kept high with cognitive puzzles. Before and after each condition measurements were conducted

Initial screening included registration of demographic characteristics and a general health questionnaire. Afterwards an activity monitor (ActivPAL™ micro, PAL Technologies, Glasgow, UK) was attached to the upper right leg for one week to characterise our study population’s average sedentary time and sleep time. Data was analysed with a modified version of the script written by Winkler et al. [25], in a similar way as previously reported [26]. Time in bed was categorised as sleep, based on sleep diaries, and reported daytime naps were excluded from sitting time as well. During four subsequent weeks, participants came to the research centre (Radboud University Medical Center) at the same time in the morning for four measurement days separated by a median of six days. This cross-over trial consisted of four measurement conditions (SIT-, BREAK-, SIT+, BREAK+), in which all participants performed all conditions (Fig. 1). Each of the four measurement days had two similar sets of cognitive and hemodynamic measurements separated by a three-hour period. A three-hour intervention period was chosen as this was enough to result in vascular changes in previous research [10], and could still reflect a real-life situation. This three-hour period differed for the four days in the interruption of sitting (SIT = no interruption, or BREAK = two-minute light-intensity walking breaks at the participant’s own pace every 30 min), and in the mental task performed (− = low mental activity, or + = high mental activity). Low mental activity constituted of watching an informative non-arousing television program called “Rail Away”, high mental activity was accomplished by making puzzles with the CogPack training software (version 9.4, Marker Software, Ladenburg, Germany).

The subjective level of mental activity was assessed three times during the sitting period with the Mental Effort Questionnaire [27]. These scores were averaged per condition, with a higher score indicating more mental activity. As expected, the SIT- and BREAK- condition scored on average -75.9 (-135.2;-16.4) points lower compared to the SIT+ and BREAK+ condition. Participants were supervised at all times to ensure adherence. The order of the conditions (SIT-, BREAK-, SIT+, BREAK+) was randomised for all participants. Participants were asked to comply with standardised diet recommendations during the 18 h preceding the measurement, including caffeine abstinence, and to avoid vigorous physical activity of at least 15 min one day prior to the measurements. Standardised breakfast consisted of bread with either jam or apple syrup, without butter, and water, and was consumed at home.

### Cognitive measurements

Multiple cognitive tests from the Test of Attentional Performance (TAP 2.3.1) battery (Psytest, Herzogenrath, Germany) were used to assess attention, executive functioning, and working memory [28], which are cognitive functions sensitive to short-term changes [29]. Participants practiced cognitive tests during the screening visit in order to reduce practice effects. A median reaction time was extracted from the alertness test to measure attention. A flexibility score was calculated based on the speed accuracy trade-off scores (SATs) of three set-shifting tasks from the flexibility test. For this measure of executive functioning, higher scores represent better executive functioning. Two levels of working memory load were measured in terms of error percentages. A detailed description of the cognitive measures can be found in Supplement 2.

### Hemodynamic measurements

Heart rate (HR) was recorded with a 3-lead electrocardiogram system (Biopac Systems inc, Goleta, CA, USA). Blood pressure (BP) was assessed continuously on the third digit of the right hand with a volume clamp-photoplethysmography device (Finapres NOVA, Finapres Medical Systems, Amsterdam, The Netherlands). The hand was kept at heart level with an arm sling. CBF velocity (CBFV) was assessed in both middle cerebral arteries (MCA) with transcranial Doppler ultrasonography (Compumedics DWL, dual 2-MHz transducer, Singen, Germany) at the temporal window, fixated in a headband (Spencer Technologies, Seattle, WA, USA). The MCA was identified based on specific waveform characteristics and insonation depth by the same sonographer on each occasion. End-tidal CO_2_ was recorded with a nasal cannula (Biopac Systems inc, Goleta, CA, USA). All these parameters were continuously recorded on a computer using AcqKnowledge 4.2.0 (Biopac Systems inc, MP160, Goleta, CA, USA).

During a five-minute measurement in seated position, baseline mean BP, CBFV, cerebrovascular resistance, and CA were assessed. Baroreflex sensitivity was measured during repeated sit-to-stand manoeuvres for five minutes (10s sitting, 10s standing). Secondary analysis additionally assessed CA during these repeated sit-to-stand manoeuvres. Cerebral vasomotor reactivity (CVMR) was measured by inducing hypocapnia (30s hyperventilation at 0.5 Hz) followed by hypercapnia (4 min of 5% CO_2_ inhalation). Protocols were similar to ones previously described [30].

### Hemodynamic data processing and analysis

All hemodynamic data were preprocessed using a semi-automated Matlab script (version 2014b, the MathWorks), transforming it to beat-to-beat data to calculate mean arterial BP and mean CBFV [30]. Cerebrovascular resistance was calculated by dividing mean arterial BP by CBFV. CA was quantified using a transfer function analysis of BP and CBFV using the CARNet Matlab script (V1) available at www.car-net.org/content/resources/tools and following guideline recommendations from the CARNet white paper [31]. This results in the parameters gain, phase, and coherence. Gain represents the amount of damping by CA on the BP oscillations, where a lower gain indicates better CA. Phase expresses the shift between CBF and BP signals due to the faster recovery of CBF changes compared to BP changes as a result of CA [32]. This is an indicator of adaptive vasoconstriction and vasodilatation, with a higher phase indicating an intact CA. Lastly, the parameter coherence can be used to identify if the gain and phase variables are reliable, as it indicates the amount of output variance explained by the input [31]. Participants with a coherence below 0.3 or visual signs of a phase wrap-around for either the very low frequency (VLF) or low frequency (LF)-domain were excluded from the analysis. To calculate the baroreflex sensitivity, a transfer function analysis on the BP and R-R interval was used around the 0.05 Hz domain, whereby a higher gain indicates higher baroreflex sensitivity [30]. For this analysis a coherence of > 0.6 was used, because the induced oscillations are expected to lead to higher coherence compared to the spontaneous oscillations in the CA analysis. CVMR (%) was defined as follows: (Max CBFV_hypercapnia_ – Min CBFV_hypocapnia_) / Mean CBFV_rest_ × 100. CVMR corrected for BP changes was calculated with the cerebrovascular conductance index (ratio of CBFV to mean arterial BP). Measurements with a change in CBFV of < 1.0 cm/s compared to baseline for either the hyper- or hypocapnia measurement were excluded. All scripts used were previously described in more detail [30].

### Statistics

A mixed model analysis was performed with separate terms for time, and the interaction between time and sit/break and mental activity respectively. Additionally, the model was corrected for the order of the first measurement condition, because randomisation resulted in 9 of the 22 participants starting with condition SIT+. Secondary analysis investigated differences in effects between sex, and antihypertensive drug use. Data were analysed using SAS statistical software (version 9.2). Two-tailed testing was used with an alpha of 0.05. Effects were reported as unstandardized B coefficients with 95% confidence intervals (CIs), characteristics as mean (SD) or percentage (n). Prior to the study, we performed a sample size calculation (α = 0.05, β = 80%) which indicated that 22 participants were needed to detect a difference of 30 (SD = 50) milliseconds in reaction time of the TAP alertness test between conditions. Cognition was chosen as the primary outcome, since it was the most distal effect, resulting in enough power to also detect changes in more proximal outcome parameters.

## Results

We included 22 cognitively healthy older adults, with a mean age of 78 years (Table 1). Most participants attended higher education (15/22), the remaining had a medium level of education. Nine of the participants were women and 50% used antihypertensive drugs. During an average week, participants were sedentary for more than ten hours/day.

**Table 1:** Participant characteristics

Variable	Mean (SD) / Percentage (n)
Age (years, (SD))	78 (5.3)
Sex (% women, (n))	41% (9)
BMI (kg/m^2^, (SD))	26 (4.0)
SBP/DBP^¥^ (mmHg, (SD))	151 / 83 (22 / 13)
MoCA (score, (SD))	26^*^ (2)
Self-reported comorbidities (%, (n))	
Myocardial infarction	18% (4)
Arterial fibrillation	5% (1)
Hypertension	41% (9)
Hypercholesterolemia	18% (4)
Diabetes	0% (0)
Arthrosis	27% (6)
Medication use (%, (n))	
Antihypertensive drugs	50% (11)
ARB	18% (4)
ACE inhibitor	23% (5)
Diuretic	18% (4)
Beta blocker	23% (5)
Calcium channel blocker	5% (1)
Statins	23% (5)
Antithrombotic agents	23% (5)
Activity pattern by activPAL^†^	
Sedentary time (hours/day, (SD))	10.3 (1.6)
Sleep time (hours/day, (SD))	8.2 (0.9)

*Abbreviations*: *BMI* Body Mass Index, *SBP* systolic blood pressure, *DBP* diastolic blood pressure, *MoCA* Montreal Cognitive Assesment, *ARB* angiotensin II receptor blocker, *ACE* angiotensin-converting-enzyme. ^¥^Blood pressure measured with an automatic oscillometric device. *Score out of a maximum of 30. †N = 21 due to one activPAL measurement with too few measurement days

### Cognitive measurements

**Fig. 2:**
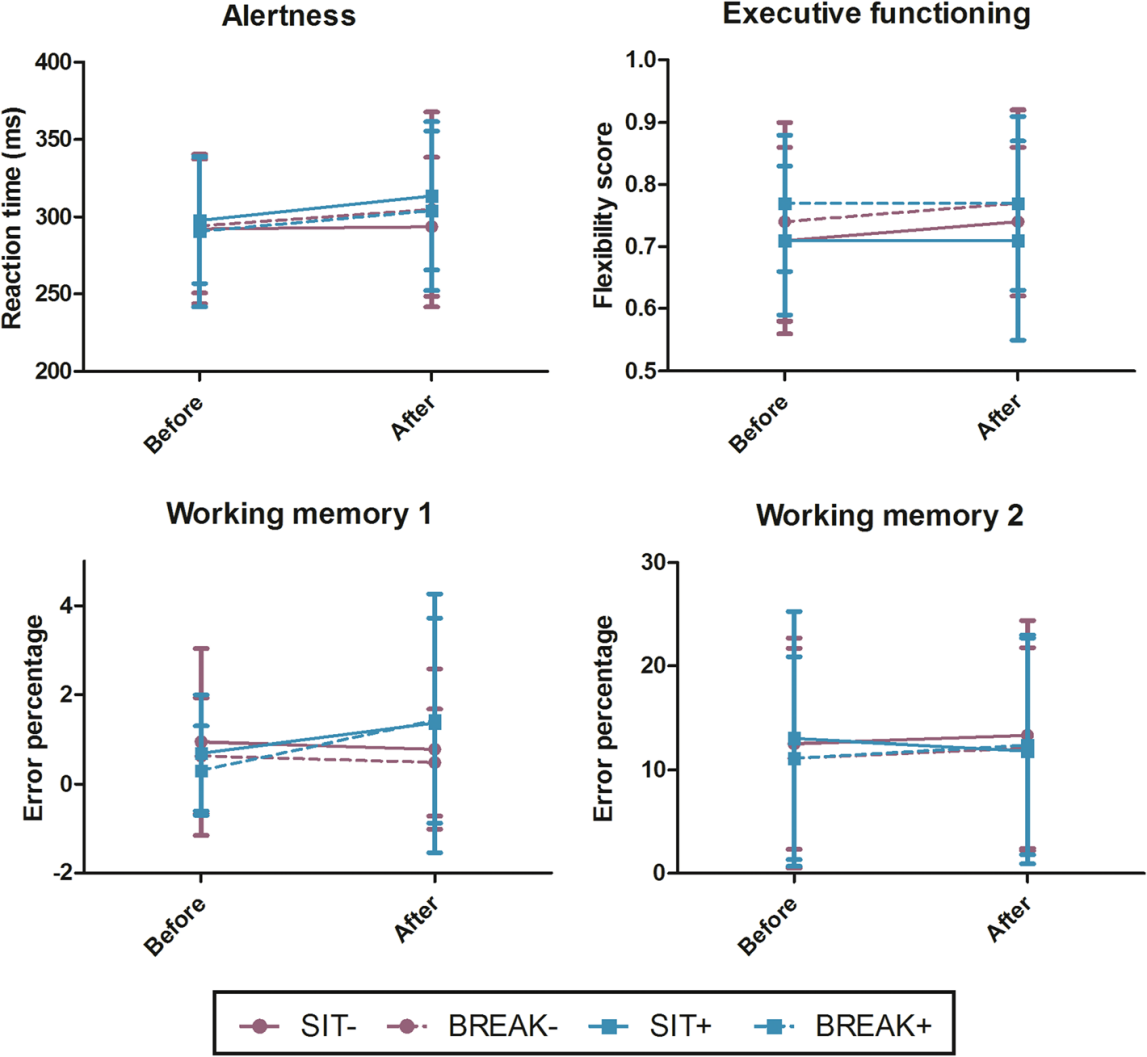
Cognitive outcome measures. Alertness reaction times, flexibility scores (higher = better), and error percentages for working memory 1 and 2 before and after the three-hour period per condition. N = 22. Purple continuous line SIT-, purple dotted line BREAK-, blue continuous line SIT+, and blue dotted line BREAK+. Mean values are shown with SDs

Reaction times during the alertness tests were higher after high mental activity (i.e. SIT+ and BREAK+) versus low mental activity (i.e. SIT- and BREAK-) (B = 8.6 (0.27;17.0), *P* = 0.04, Fig. 2). For working memory 1, higher error percentages were found after high mental activity (B = 0.79 (0.16;1.41), *P* = 0.01). No significant differences were found for executive functioning and working memory 2 across time or between conditions.

### Hemodynamic measurements

**Fig. 3:**
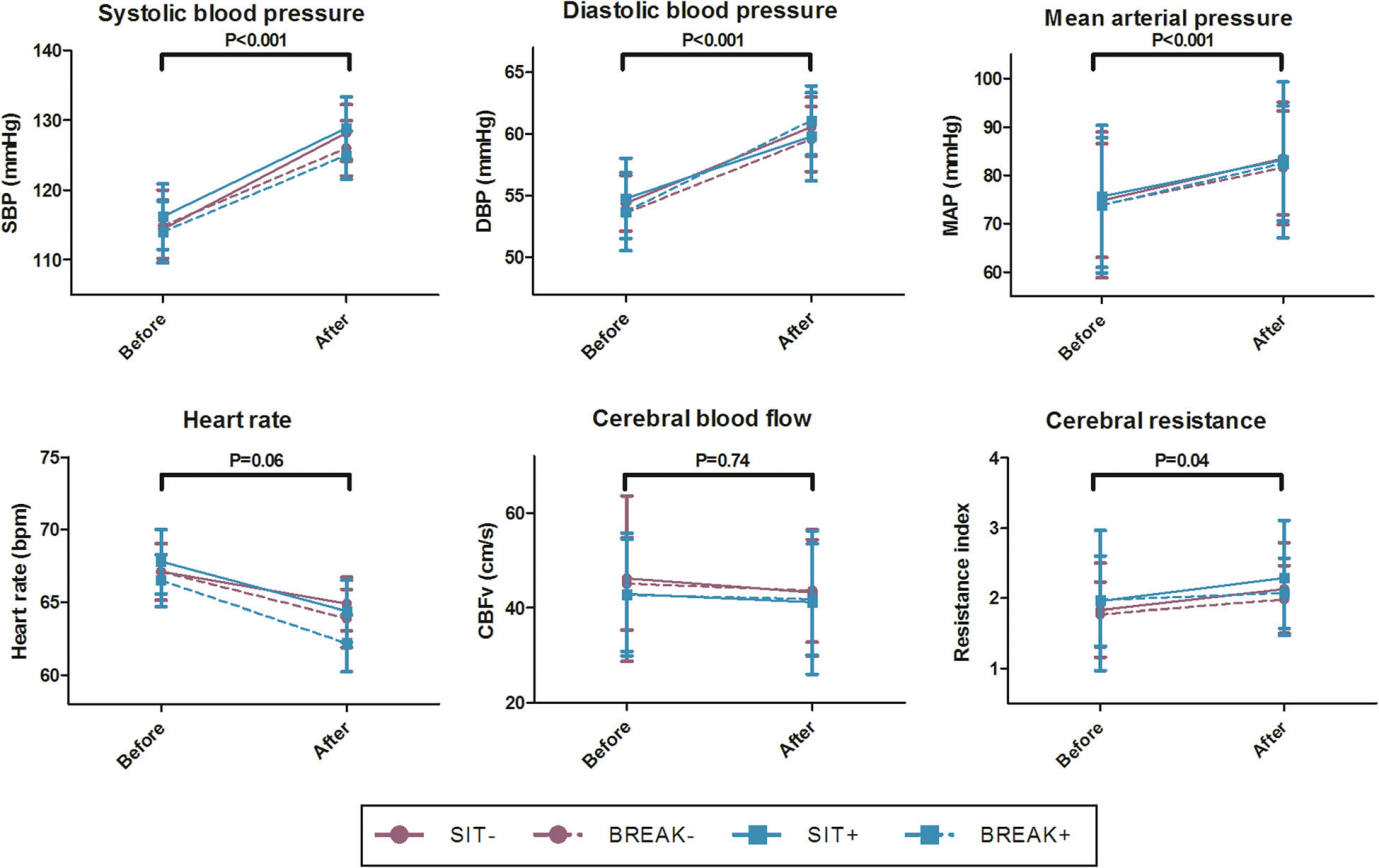
Hemodynamic outcome measures. Systolic, diastolic and mean arterial pressure measured by Finapres NOVA, heart rate, cerebral blood flow, and cerebral resistance before and after the three-hour period per condition. Purple continuous line SIT-, purple dotted line BREAK-, blue continuous line SIT+, and blue dotted line BREAK+. Mean values are shown with SDs. P-values represent the time effect over three hours. For SBP, DBP, Mean arterial BP, and HR N = 20 (except for SIT+ N = 19). For CBF and CVR N = 16 for SIT-, N = 15 for BREAK- and BREAK+, N = 17 for SIT+

BP significantly increased over time (B = 8.6 mmHg in mean arterial pressure (5.0;12.1), *P* < 0.001), which did not differ between conditions (Fig. 3). Stratification showed that this increase was seen in both people with and without antihypertensive medication, as well as in males and females separately (data not shown). BP variability, expressed as the standard deviation during the five minute baseline measurement, did not change over time or between conditions (data not shown). No change was found in CBFV over time (B = -0.6 (-4.1;2.9), *P* = 0.74). The three-hour intervention was associated with a significant increase in cerebrovascular resistance (B = 0.23 (0.01;0.45), *P* = 0.04). There was no significant interaction with antihypertensive drug use or sex (data not shown). No differences were found in changes in CBFV or cerebrovascular resistance between interventions either. Additionally, no effects of the walking breaks (i.e. SIT−/+ vs BREAK−/+), independent from mental activity, were found on any of the outcome parameters.

Baroreflex sensitivity did not change over time or between conditions (Supplement 3). In the assessment of CA no differences in gain, normalised gain or phase were observed for the VLF and LF domain between the different conditions over time (Table 2). However, VLF phase increased significantly after three hours (B = 11.0 (3.52;18.46), *P* = 0.01) in all conditions. Transfer function analysis on the repeated sit-stand measurement (Supplement 4) showed a decrease in normalised gain over time (B = -0.19 (-0.35;-0.03), *P* = 0.03) in all conditions.

**Table 2:** Cerebral autoregulation parameters per condition and time point

	SIT-		BREAK-		SIT+		BREAK+	
	Before	After	Before	After	Before	After	Before	After
**N VLF**	11	10	12	9	12	12	10	11
**Gain**_**VLF**_**, cm s**^**−1**^**mmHg**^**−1**^	0.60 (0.34)	0.43 (0.10)	0.54 (0.13)	0.49 (0.13)	0.47 (0.12)	0.47 (0.21)	0.50 (0.19)	0.46 (0.14)
**Gain**_**norm-VLF**_**, % mmHg**^**−1**^	1.3 (0.61)	1.1 (0.26)	1.3 (0.29)	1.1 (0.35)	1.2 (0.36)	1.1 (0.34)	1.2 (0.29)	1.2 (0.41)
**Phase**_**VLF**_, degrees^*^	42.8 (14.6)	54.4 (16.2)	50.8 (19.2)	61.0 (16.4)	43.7 (17.6)	54.7 (18.7)	45.0 (14.6)	51.5 (14.2)
**N LF**	11	13	15	14	14	13	11	12
**Gain**_**LF**_**, cm s**^**−1**^**mmHg**^**−1**^	0.63 (0.29)	0.53 (0.16)	0.70 (0.19)	0.68 (0.19)	0.67 (0.26)	0.60 (0.23)	0.63 (0.28)	0.60 (0.19)
**Gain**_**norm-LF**_**, % mmHg**^**−1**^	1.6 (0.59)	1.4 (0.34)	1.6 (0.34)	1.6 (0.38)	1.6 (0.37)	1.6 (0.43)	1.5 (0.40)	1.4 (0.43)
**Phase**_**LF**_**, degrees**	25.6 (8.0)	32.7 (11.0)	29.7 (9.2)	25.8 (11.1)	31.6 (12.5)	28.4 (13.1)	33.0 (12.1)	27.9 (14.4)

Values represent the mean (SD). *Abbreviations*: *VLF* very-low-frequency, *LF* low-frequency, *norm* normalised, *N* number of participants. ^*^Phase of the VLF increased significantly after three hours (11.0 (3.52;18.46), P = 0.01) in all conditions

Hypocapnia resulted in a mean CBFV drop of 28% compared to baseline normocapnia, and there was a 25% increase in CBFV during hypercapnia. This CVMR did not change over time (B = 1.8 (-4.0;7.5), *P* = 0.54) or between conditions (Supplement 5).

## Discussion

This cross-over trial in older adults identified that three hours of sitting did not have an effect on cognitive functioning. In line with this, no indications for short-term SB-induced effects were seen on CBFV, CA, baroreflex sensitivity, or CVMR. However, increases in BP and cerebrovascular resistance were present. Importantly, we found no evidence for an impact of breaking-up the three-hour sitting period by two-minute light-intensity walks, whilst also no differential effect was found between low versus high mental activity on cerebrovascular hemodynamics.

After three hours of sitting we did not observe any differences in cognitive performance. A previous pilot study in adults found similar results, where cognitive function was not different between a five-hour uninterrupted sitting period, and five hours with three minute walking breaks [33]. Likewise, even after a sedentary intervention that entailed a full week of sitting no differences in cognitive function were found, but this concerned young adults [34]. We do not think our findings were the result of test insensitivity, because we did show that the test battery we used was responsive to changes: after three hours of mental activity, an increase in reaction time (B = 8.6 (0.27; 17.0), *P* = 0.04) and increase in error percentage (B = 0.79 (0.16; 1.41), *P* = 0.01) was found. The subjective assessment already showed that participants judged the SIT+ and BREAK+ conditions cognitively more challenging compared to the SIT- and BREAK- conditions. This demonstrates that the differentiation in mental activity between the conditions was successful, but also that the mental activity during the three-hour period did not have a stimulating effect on cognitive performance. Contrary, vigilance decrements were observed. A vigilance decrement is characterised by increases in reaction times and decreases in correct responses over time. Theory suggests these decrements are potentially explained by high cognitive demands [35]. The puzzles made during the sitting period could have, in line with this theory, depleted the cognitive resource pool, subsequently resulting in worse cognitive performance after three hours compared to the low mental activity conditions.

The absence of an SB-induced effect on cognitive performance is therefore more likely explained by the unaffected CBFV levels, than by test insensitivity. Cognitive functions with adequate responsiveness are shown to be affected by CBF levels [36], however our CBFV levels showed no change over three hours of sitting either. This is in contrast to previous studies that found reduced CBFV after uninterrupted sitting [13, 14]. However, both groups were younger and the sitting period was longer (four and eight hours). As CBF reduces with age [37], the ability to reveal an absolute decline in CBFV diminishes, which may partly explain our null finding. At the same time, this stable CBF points towards preserved CA, especially given the observed increase in BP. In order to prevent the increase in BP from affecting CBF levels, cerebrovascular resistance also has to increase to keep CBF stable. This increase in cerebrovascular resistance in itself is thus an acute reaction part of the CA on the SB-induced increase in BP to maintain a stable CBF. However, the long-term effects of repeated increases in cerebral resistance can have negative consequences for the cerebral vasculature. Specifically, an increase in resistance contributes to vascular remodelling and narrowing of the vessel lumen [38]. Through these changes an increased risk of hypoperfusion arises that might contribute to the progression of cognitive decline [39]. Therefore, despite the absence of short-term effects on both CBF levels and cognition, the chronic exposure to repeated prolonged sitting bouts may still have a negative long-term effect on the two.

These results thus show that after three hours of sitting regulatory mechanisms such as CA and the baroreflex are still preserved. Even though CA appeared slightly lower in our older study population at baseline (mean VLF phase 40–50 degrees) compared to young adults (mean VLF phase 56.7 degrees) [40], CA indeed did not decline over the three-hour period. Extreme examples of inactivity during short-term space flights showed preserved CA as well; potential increases were even observed [41]. Over the 3-h period we similarly noted small improvements in the phase and gain parameters. This may support the concept that CA represents a robust regulation mechanism, which is substantiated by the fact that it even seems preserved in Alzheimer’s disease despite the cerebrovascular pathology also associated with that disease [42]. Moreover, CA may be independent from endothelial function, as the inhibition of nitric oxide synthase did not affect CA in young adults [43]. This may explain why CA is unlikely to be affected in the short-term by uninterrupted sitting, and may in fact compensate for potential (short-term) alternations in endothelial function. Regarding the second regulatory mechanism, the baroreflex, microgravity studies indicated blunting of its sensitivity [19]. This is contrary to our observation of preserved baroreflex sensitivity after three hours of sitting. In our study, the sitting period did not result in a reduction in BP variability. We speculate that this preserved variability in BP kept baroreceptors stimulated, also maintaining the sensitivity of this regulatory mechanism.

In contrast to the indications of unaffected cognitive function, CBF, and regulatory mechanisms of CA and baroreflex, BP increased over time. Previous research already indicated increases in BP after periods of uninterrupted sitting [44]. The hypothesis underlying this effect is the low metabolic demand during uninterrupted sitting [44], subsequently leading to constriction of the capillary blood vessels, causing reduced blood flow. Consequently, a reduction in shear stress and stimulation of vasoconstrictors is observed, resulting in increased peripheral resistance and higher BP [44]. This is a more probable explanation for the rise in BP than a time-effect caused by the circadian rhythm, as this peaks mostly between 6 and 10 am in the early morning [45]. However, the mean increase of 8.6 mmHg in mean arterial BP over time, across conditions, in our study is more pronounced than the general trend of BP over time observed in previous literature [46]. This could partly be explained by postprandial BP lowering that was observed in earlier studies that started with a meal just before the start of the experiments. In our study, no meal in the lab was provided. Participants ate a standardised breakfast at home, and postprandial effects would have ended by the time the pre-intervention measurements took place more than 1.5 h later [47]. Another explanation for the 8.6 mmHg increase in BP is that our study population is older compared to previous studies. Older age is typically linked to impaired vascular function, which is subsequently associated with exaggerated vascular responses to vasoactive stimuli [48]. Indeed, our subjects demonstrated a pulse pressure (SBP - DBP; i.e. a proxy of arterial stiffness) of 68.8 mmHg, which was markedly higher than in previous work (i.e. 45.4 mmHg in healthy younger adults (mean age 54) [49]. This may contribute to the high SB-induced increase in BP in our study. This suggests that the impact of sitting may be exaggerated in the presence of impaired vascular health, although future work is required to better understand this potential link.

In contrast to previous work [13, 14, 46], the increases in BP and cerebral resistance were not mitigated by two-minute walks every 30 min. These breaks were thought to counteract the effects of sitting by increasing metabolic demand within muscles, leading to an increase in vasodilators that increase flow and shear stress [44], subsequently reducing peripheral resistance and BP. Nevertheless, no decrease in BP was found in our group after two-minute walking breaks, which is an important notion for interventions aiming to reduce the effects of SB. The high pulse pressure we found is thought to reflect increased vascular stiffness in this group, which might result in a reduced vasodilatory capacity [50]. Therefore, the light-intensity walking breaks might not provide sufficient stimulation in our older population to counteract the increase in BP. Studies that found walking breaks to prevent an increase in BP were conducted in healthy young adults [46]. Possibly, older adults not only show an increased response to prolonged bouts of sitting, but also need to break-up these bouts with activities that have a higher intensity or at a higher frequency to combat the negative effects.

### Strengths and limitations

Due to the cross-over design the influence of inter-individual differences was minimised. By standardising the mental task, variation in activities during the sitting period was reduced and mentally active and inactive SB could be compared in their short-term effects on cognition and hemodynamical responses. In contrast to the screening instruments often used, the sensitive cognitive measures made it possible to detect subtle changes in cognitive performance. Combining the cognitive measures with the hemodynamic measurements enabled us to see the impact of uninterrupted sitting on brain physiology and its potential consequences in an older population that is of greatest interest due to the ageing population. However, there are also some limitations that should be kept in mind, especially to prevent overinterpreting of the null findings presented in this study. For example, the assessment of CBFV with transcranial Doppler ultrasonography has some downfalls, especially in older adults. The quality is not always optimal due to the thickness of the temporal bone [51], which even resulted in two participants where no signal could be acquired at all. For the remaining participants in all the conditions approximately 4 to 5 of the signals could not be analysed due to limited signal quality or inadequate comparison between the before and after-measurement. This is also the result of removing the probe at the start of the three-hour sitting period. The re-attachment of the probe for the post-intervention measurement could have resulted in the insonation of a different part of the middle cerebral artery. However, the same researcher was always in charge of attaching the probes and finding signal in a similar way. Additionally, during pre-processing we checked and confirmed data quality. Continuous measurements of BP and CBFV during the three-hour period would have been ideal, but lead to discomfort (e.g. headache from prolonged probe fixation) that would have affected our measurements and would have resulted in significant dropouts. The lower number of participants with valid data could however have resulted in insufficient power for the hemodynamical analyses which should be kept in mind. Especially the heterogeneity in vascular disorders and potentially in cognitive status, could have influenced the results in our small sample. At the same time, the specific characteristics of this sample, such as relative high education, reduce the generalisability of our results. Furthermore, it should be noted that the MCA that was measured here perfuses approximately 70% of the brain, and not all brain areas, for example the posterior and anterior areas. Therefore, the results on CBFV should not be generalised to the whole brain circulation. Lastly, the intensity of the activity breaks used to interrupt the sitting bouts were not registered.

## Conclusions

Our cross-over study showed that following three hours of uninterrupted sitting cognitive performance was not affected. In line with this, no effects were seen on cerebral blood flow velocity and the regulatory mechanisms of cerebral autoregulation and the baroreflex. However, blood pressure and cerebrovascular resistance increased in these older adults after three hours. Mental activity or two-minute light-intensity walking breaks did not mitigate these increases. Despite the absence of short-term effects on cognitive performance, this does not preclude sedentary behaviour from having potential negative effects on the brain over a longer period. Possibly, chronic exposure to repeated increases in blood pressure and cerebrovascular resistance associated with prolonged sitting might negatively affect the brain and cognitive functioning. More research on the long-term effects and preventive strategies is therefore needed as the impact of prolonged sitting on the vasculature seems more pronounced and more difficult to counterbalance in older adults, potentially due to their age-related reduction in vascular function.

## Supplementary information

**Additional file 1 : Supplement 1** Flow chart.

**Additional file 2 : Supplement 2** Detailed description of the cognitive tasks.

**Additional file 3 : Supplement 3** Baroreflex sensitivity results repeated sit-stands.

**Additional file 4 : Supplement 4** Cerebral autoregulation results repeated sit-stands.

**Additional file 5 : Supplement 5** Cerebrovascular reactivity results.

## Data Availability

The datasets generated and/or analysed during the current study are not publicly available due to data protection regulations, but are accessible at the corresponding author on reasonable request.
