# Supplementary material for: The short-term effects of sedentary behaviour on cerebral hemodynamics and cognitive performance in older adults: a cross-over design on the potential impact of mental and/or physical activity
Source: Alzheimers Res Ther. 2020 Jun 22;12:76. doi: 10.1186/s13195-020-00644-z (PMC7310280; doi:10.1186/s13195-020-00644-z)
Supplement: Supplementary file 1 — Additional file 1 : Supplement 1 Flow chart. [file 13195_2020_644_MOESM1_ESM.docx]

**Supplement 1 – Flow chart**
