# Supplementary material for: The short-term effects of sedentary behaviour on cerebral hemodynamics and cognitive performance in older adults: a cross-over design on the potential impact of mental and/or physical activity
Source: Alzheimers Res Ther. 2020 Jun 22;12:76. doi: 10.1186/s13195-020-00644-z (PMC7310280; doi:10.1186/s13195-020-00644-z)
Supplement: Supplementary file 2 — Additional file 2 : Supplement 2 Detailed description of the cognitive tasks. [file 13195_2020_644_MOESM2_ESM.docx]

**Supplement 2 – Detailed description of the cognitive function measurements**

Multiple tests from the Test of Attentional Performance (TAP 2.3.1) battery (Psytest, Herzogenrath, Germany) were used to assess different cognitive functions: attention, executive functioning, and working memory. Attention was measured with the Alertness subtest that consists of two parts in which the participants had to react as quickly as possible to a cue (cross presented in the middle of the screen) preceded by no (intrinsic) or an acoustic (phasic) warning signal. Median reaction times of the intrinsic and phasic alertness tests were averaged. To measure executive functioning, the Flexibility subtest was used, in which a round and angular stimulus were presented simultaneously on the screen in each trial, to which the participant had to respond in accordance with a rule. The test consists of three conditions. The first two conditions are simple set-shifting tasks, in which the participants had to indicate as quickly as possible on which side (left or right) the angular (condition 1) or the round (condition 2) stimulus was presented. In the third condition, the target stimuli were alternated (angular – round – angular – ...). A flexibility measure was computed by calculating the speed accuracy trade-off score (SAT) based on median reaction time and accuracy percentage for the angular-condition, round-condition and alternating condition. The SAT of the alternating condition was divided by the averaged square-condition and round-condition SATs to obtain a measure of executive functioning. Lastly, the subtest Working Memory was used to measure working memory with two load conditions in which participants were shown a sequence of numbers, presented one by one. For the first test (Working Memory 1) participants had to react to a stimulus if it was similar to the previous number (low load), for the second test (Working Memory 2) the number should be similar to the second-last item (higher load). The percentage errors and omissions made in working memory 1 and 2 were averaged for both tasks respectively due to the difference in difficulty level.
