# Supplementary material for: The short-term effects of sedentary behaviour on cerebral hemodynamics and cognitive performance in older adults: a cross-over design on the potential impact of mental and/or physical activity
Source: Alzheimers Res Ther. 2020 Jun 22;12:76. doi: 10.1186/s13195-020-00644-z (PMC7310280; doi:10.1186/s13195-020-00644-z)
Supplement: Supplementary file 3 — Additional file 3 : Supplement 3 Baroreflex sensitivity results repeated sit-stands. [file 13195_2020_644_MOESM3_ESM.docx]

**Supplement 3 – Baroreflex sensitivity results repeated sit-stands**

Mean Arterial Pressure (mmHg) during repeated sit-stands

| **Condition** | **Time** | **Mean** | **SD** | **N** |
| --- | --- | --- | --- | --- |
| SIT- | Before | 128.65 | 19.4 | 20 |
| SIT- | After | 141.89 | 19.7 | 20 |
| BREAK- | Before | 129.20 | 23.2 | 20 |
| BREAK- | After | 138.24 | 20.8 | 20 |
| SIT+ | Before | 128.56 | 21.8 | 20 |
| SIT+ | After | 139.96 | 20.0 | 19 |
| BREAK+ | Before | 126.59 | 20.6 | 17 |
| BREAK+ | After | 140.39 | 18.3 | 18 |

| **Effect** | **Estimate** | **P-value** |
| --- | --- | --- |
| Intercept | 127.54 (119.06 ; 136.03) | <0.001* |
| Time | 12.18 (7.04 ; 17.32) | <0.001* |
| Time×Stand | -0.96 (-6.18 ; 4.27) | 0.72 |
| Time×Mental | -0.76 (-5.95 ; 4.44) | 0.77 |
| Order | 3.63 (-0.76 ; 8.02) | 0.10 |

***^*^****Indicates statistical significance (P<0.05).*

RR-interval during repeated sit-stands

| **Condition** | **Time** | **Mean** | **SD** | **N** |
| --- | --- | --- | --- | --- |
| SIT- | Before | 0.84 | 0.11 | 20 |
| SIT- | After | 0.84 | 0.11 | 20 |
| BREAK- | Before | 0.86 | 0.13 | 20 |
| BREAK- | After | 0.86 | 0.12 | 20 |
| SIT+ | Before | 0.86 | 0.12 | 20 |
| SIT+ | After | 0.85 | 0.13 | 19 |
| BREAK+ | Before | 0.87 | 0.10 | 17 |
| BREAK+ | After | 0.86 | 0.11 | 18 |

| **Effect** | **Estimate** | **P-value** |
| --- | --- | --- |
| Intercept | 0.86 (0.81 ; 0.91) | <0.001* |
| Time | -0.01 (-0.03 ; 0.01) | 0.27 |
| Time×Stand | 0.01 (-0.01 ; 0.03) | 0.48 |
| Time×Mental | -0.00 (-0.02 ; 0.02) | 0.90 |
| Order | -0.01 (-0.03 ; 0.01) | 0.34 |

***^*^****Indicates statistical significance (P<0.05).*

Baroreflex Gain during repeated sit-stands

| **Condition** | **Time** | **Mean** | **SD** | **N** |
| --- | --- | --- | --- | --- |
| SIT- | Before | 2.94 | 1.5 | 12 |
| SIT- | After | 2.98 | 1.7 | 14 |
| BREAK- | Before | 2.86 | 1.5 | 17 |
| BREAK- | After | 3.20 | 1.3 | 15 |
| SIT+ | Before | 2.86 | 1.3 | 15 |
| SIT+ | After | 3.38 | 1.4 | 17 |
| BREAK+ | Before | 3.57 | 1.7 | 13 |
| BREAK+ | After | 3.27 | 1.1 | 12 |

| **Effect** | **Estimate** | **P-value** |
| --- | --- | --- |
| Intercept | 2.92 (2.31 ; 3.54) | <0.001* |
| Time | 0.08 (-0.37 ; 0.52) | 0.74 |
| Time×Stand | -0.03 (-0.48 ; 0.42) | 0.90 |
| Time×Mental | 0.14 (-0.31 ; 0.58) | 0.55 |
| Order | 0.16 (-0.23 ; 0.55) | 0.42 |

***^*^****Indicates statistical significance (P<0.05).*
