# Supplementary material for: The short-term effects of sedentary behaviour on cerebral hemodynamics and cognitive performance in older adults: a cross-over design on the potential impact of mental and/or physical activity
Source: Alzheimers Res Ther. 2020 Jun 22;12:76. doi: 10.1186/s13195-020-00644-z (PMC7310280; doi:10.1186/s13195-020-00644-z)
Supplement: Supplementary file 4 — Additional file 4 : Supplement 4 Cerebral autoregulation results repeated sit-stands. [file 13195_2020_644_MOESM4_ESM.docx]

**Supplement 4 – Cerebral autoregulation results repeated sit-stands**

Gain very-low frequency during repeated sit-stands

| **Condition** | **Time** | **Mean** | **SD** | **N** |
| --- | --- | --- | --- | --- |
| SIT- | Before | 0.76 | 0.43 | 8 |
| SIT- | After | 0.50 | 0.13 | 8 |
| BREAK- | Before | 0.62 | 0.14 | 10 |
| BREAK- | After | 0.63 | 0.23 | 10 |
| SIT+ | Before | 0.63 | 0.27 | 8 |
| SIT+ | After | 0.61 | 0.22 | 8 |
| BREAK+ | Before | 0.61 | 0.16 | 9 |
| BREAK+ | After | 0.70 | 0.30 | 11 |

| **Effect** | **Estimate** | **P-value** |
| --- | --- | --- |
| Intercept | 0.67 (0.55 ; 0.80) | <0.001* |
| Time | -0.10 (-0.20 ; 0.00) | 0.05 |
| Time×Stand | 0.05 (-0.06 ; 0.15) | 0.37 |
| Time×Mental | 0.07 (-0.02 ; 0.17) | 0.14 |
| Order | -0.07 (-0.16 ; 0.03) | 0.95 |

***^*^****Indicates statistical significance (P<0.05).*

Normalised gain very-low frequency during repeated sit-stands

| **Condition** | **Time** | **Mean** | **SD** | **N** |
| --- | --- | --- | --- | --- |
| SIT- | Before | 1.54 | 0.43 | 8 |
| SIT- | After | 1.25 | 0.30 | 8 |
| BREAK- | Before | 1.64 | 0.36 | 10 |
| BREAK- | After | 1.67 | 0.35 | 10 |
| SIT+ | Before | 1.50 | 0.53 | 8 |
| SIT+ | After | 1.43 | 0.31 | 8 |
| BREAK+ | Before | 1.57 | 0.32 | 9 |
| BREAK+ | After | 1.50 | 0.30 | 11 |

| **Effect** | **Estimate** | **P-value** |
| --- | --- | --- |
| Intercept | 1.63 (1.45 ; 1.881) | <0.001* |
| Time | -0.19 (-0.35 ; -0.03) | 0.03* |
| Time×Stand | 0.16 (-0.01 ; 0.33) | 0.06 |
| Time×Mental | 0.01 (-0.16 ; 0.17) | 0.95 |
| Order | -0.14 (-0.29 ; 0.02) | 0.09 |

***^*^****Indicates statistical significance (P<0.05).*

Phase very-low frequency during repeated sit-stands

| **Condition** | **Time** | **Mean** | **SD** | **N** |
| --- | --- | --- | --- | --- |
| SIT- | Before | 51.3 | 17.2 | 8 |
| SIT- | After | 55.0 | 19.8 | 8 |
| BREAK- | Before | 45.8 | 17.3 | 10 |
| BREAK- | After | 51.3 | 10.7 | 10 |
| SIT+ | Before | 54.8 | 10.7 | 8 |
| SIT+ | After | 51.5 | 10.2 | 8 |
| BREAK+ | Before | 40.2 | 16.6 | 9 |
| BREAK+ | After | 48.9 | 17.0 | 11 |

| **Effect** | **Estimate** | **P-value** |
| --- | --- | --- |
| Intercept | 48.7 (41.5 ; 55.9) | <0.001* |
| Time | 5.4 (-1.2 ; 12.1) | 0.11 |
| Time×Stand | -4.6 (-11.4 ; 2.2) | 0.18 |
| Time×Mental | -1.4 (-8.0 ; 5.3) | 0.69 |
| Order | -1.0 (-7.3 ; 5.4) | 0.76 |

***^*^****Indicates statistical significance (P<0.05).*
