# Supplementary material for: The short-term effects of sedentary behaviour on cerebral hemodynamics and cognitive performance in older adults: a cross-over design on the potential impact of mental and/or physical activity
Source: Alzheimers Res Ther. 2020 Jun 22;12:76. doi: 10.1186/s13195-020-00644-z (PMC7310280; doi:10.1186/s13195-020-00644-z)
Supplement: Supplementary file 5 — Additional file 5 : Supplement 5 Cerebrovascular reactivity results. [file 13195_2020_644_MOESM5_ESM.docx]

**Supplement 5 – Cerebrovascular reactivity results**

Characteristics cerebral vasomotor reactivity

| **Condition** | **Time** | **Rest** | **Hypocapnia** | | **Hypercapnia** | | **N** |
| --- | --- | --- | --- | --- | --- | --- | --- |
|  |  | **Mean CBFV** | **Min CBFV** | **% change** | **Max CBFV** | **% change** |  |
| SIT- | Before | 44.5 (15.3) | 31.0 (10.1) | -29.9 | 55.9 (13.7) | 29.8 | 10 |
| SIT- | After | 46.1 (14.5) | 33.6 (11.2) | -26.8 | 58.4 (16.2) | 28.0 | 11 |
| BREAK- | Before | 45.6 (11.2) | 33.2 (8.9) | -27.2 | 56.8 (13.4) | 25.2 | 16 |
| BREAK- | After | 43.4 (11.1) | 29.9 (8.6) | -31.3 | 54.5 (15.4) | 25.0 | 16 |
| SIT+ | Before | 48.6 (13.1) | 35.9 (9.2) | -25.8 | 59.9 (18.7) | 22.2 | 8 |
| SIT+ | After | 46.5 (14.6) | 32.0 (8.6) | -29.9 | 55.2 (17.8) | 18.6 | 10 |
| BREAK+ | Before | 46.3 (12.1) | 34.3 (8.8) | -25.4 | 58.2 (18.2) | 24.5 | 13 |
| BREAK+ | After | 44.4 (11.6) | 32.2 (8.7) | -27.5 | 55.3 (16.5) | 23.9 | 10 |

*Mean, minimal and maximal cerebral blood flow velocity (CBFV) in cm/s for the rest, hypocapnia and hypercapnia measurements respectively with standard deviations. Percentage change has been calculated based on mean CBFV of the rest measurement.*

Cerebral vasomotor reactivity based on flow velocity

| **Condition** | **Time** | **Mean** | **SD** | **N** |
| --- | --- | --- | --- | --- |
| SIT- | Before | 58.9 | 28.9 | 10 |
| SIT- | After | 55.3 | 13.6 | 11 |
| BREAK- | Before | 51.5 | 11.2 | 16 |
| BREAK- | After | 56.3 | 14.7 | 16 |
| SIT+ | Before | 47.9 | 10.1 | 8 |
| SIT+ | After | 48.1 | 9.3 | 10 |
| BREAK+ | Before | 49.2 | 12.9 | 13 |
| BREAK+ | After | 49.3 | 8.2 | 10 |

*CVMR has been calculated as follows: (Max CBFV_hypercapnia_ – Min CBFV_hypocapnia_) / Mean CBFV_rest_ * 100%.*

| **Effect** | **Estimate** | **P-value** |
| --- | --- | --- |
| Intercept | 52.2 (45.5 ; 59.0) | <0.001* |
| Time | 1.8 (-4.0 ; 7.5) | 0.54 |
| Time×Stand | 1.6 (-4.2 ; 7.4) | 0.59 |
| Time×Mental | -2.6 (-8.5 ; 3.3) | 0.39 |
| Order | -2.6 (-7.9 ; 2.7) | 0.33 |

***^*^****Indicates statistical significance (P<0.05).*

Cerebral vasomotor reactivity based on cerebrovascular conductance index

| **Condition** | **Time** | **Mean** | **SD** | **N** |
| --- | --- | --- | --- | --- |
| SIT- | Before | 34.3 | 21.9 | 10 |
| SIT- | After | 32.8 | 11.5 | 11 |
| BREAK- | Before | 28.2 | 16.8 | 16 |
| BREAK- | After | 34.4 | 14.2 | 16 |
| SIT+ | Before | 21.6 | 11.8 | 8 |
| SIT+ | After | 31.2 | 12.2 | 10 |
| BREAK+ | Before | 25.4 | 10.3 | 13 |
| BREAK+ | After | 29.3 | 12.9 | 10 |

*Corrected CVMR (%) has been calculated as follows: (CVCi_hypercapnia_ – CVCi_hypocapnia_) / CVCi_rest_ × 100.*

| **Effect** | **Estimate** | **P-value** |
| --- | --- | --- |
| Intercept | 28.9 (22.8 ; 35.1) | <0.001* |
| Time | 5.9 (-0.9 ; 12.8) | 0.09 |
| Time×Stand | -1.8 (-8.7 ; 5.0) | 0.60 |
| Time×Mental | -2.3 (-9.3 ; 4.7) | 0.51 |
| Order | -1.5 (-7.6 ; 4.7) | 0.64 |

***^*^****Indicates statistical significance (P<0.05).*
